# Looking at both sides of algorithmic control and employee well-being: a job demands−resources model

**DOI:** 10.3389/fpsyg.2026.1736349

**Published:** 2026-01-21

**Authors:** Lu Zhang, Xuehang Ling, Chen Yang

**Affiliations:** 1 Department of Business Administration, Guizhou University of Finance and Economics, Guiyang, China; 2 School of Management, Chongqing University of Technology, Chongqing, China

**Keywords:** algorithmic control, feedback quality, role clarity, time pressure, well-being, work overload

## Abstract

**Objectives:**

Drawing on the job demands-resources (JD-R) theory, this study aims to analyze the impact of algorithmic control on the well-being of delivery drivers by focusing on the mediating role of job demands (work overload and time pressure) and job resources (feedback quality and role clarity).

**Methods:**

This study obtained three-wave data from 435 delivery drivers and examined the hypotheses using structural equation modelling.

**Results:**

The results indicated that algorithmic control reduced delivery drivers’ well-being by increasing the job demands (time pressure and work overload). In addition, algorithmic control enhanced delivery drivers’ well-being by increasing their level of job resources (feedback quality and role clarity).

**Conclusion:**

Integrating job demands-resources (JD-R) theory, this study provides a more balanced view of how algorithmic control influences well-being by demonstrating the opposing mediating roles of job demands (work overload and time pressure) and job resources (feedback quality and role clarity).

## 1 Introduction

According to data from the All-China Federation of Trade Unions, the number of food delivery drivers increased from 6 million in 2020 to over 10 million in 2024. Given the growing number of food delivery drivers in the workforce, platform firms are now using algorithmic control to improve the work efficiency and service quality of their drivers. Algorithmic control refers to the management practice of using intelligent algorithms and advanced digital technology to supervise and guide food-delivery driver behaviors (Deng et al., 2025; Parent-Rocheleau and Parker, 2022). As algorithmic control has reconfigured the production relationship among platforms, customers, and food delivery drivers, this has created a research hotspot focusing on how algorithmic control affects driver work outcomes (Wood et al., 2019; Parent-Rocheleau and Parker, 2022). Researchers have begun to explore the impact of algorithmic control on employees’ well-being because employees are also stakeholders in the organizations for which they work, with their well-being playing a crucial role with regard to both their own health and organizational performance (Zhang et al., 2022; Zayid et al., 2024).

The mainstream view of the extant literature is that algorithmic control can reduce employee well-being (Zayid et al., 2024; Kinowska and Sienkiewicz, 2023). Nevertheless, a contrasting view suggests that algorithmic control is beneficial to the employee work well-being by enhancing their fairness perception (Deng et al., 2025). Given these contradictory findings, the relationship between algorithmic control and well-being appears to be more complex than one might expect. Therefore, this research aims to examine the double-edged sword effects of algorithmic control on delivery drivers’ well-being and seek an effective way to harness the positive impact of algorithmic control and address its negative effects.

To address this issue, following Caesens et al. (2017) and Puranik et al. (2021), this study considered job satisfaction and emotional exhaustion as important dimensions of well-being, and examined a dual pathway model to explore the double-edged sword effects of algorithmic control on employee well-being based on the job demands-resources (JD-R) theory. JD-R theory suggests that management practices can indirectly affect employee well-being through perceived job resources and demands (Bakker et al., 2023). As algorithmic control is an important managerial practice (Wu et al., 2023), JD-R theory can be used to explain the effects of algorithmic control on the well-being of food delivery drivers experiencing increased job demands and resources. Specifically, we focused on how algorithmic control influences the job resources relating to feedback quality and role clarity, which we considered as mediators of the positive relationship between algorithmic control and food-delivery drivers’ well-being. We also focused on how algorithmic control impacts the job demands of time pressure and work overload, which we considered as intermediaries of the negative relationship between algorithmic control and food-delivery drivers’ well-being.

This study contributes to the existing literature in this field in several ways. First, pervious researches have either focused on the negative effect of algorithmic control on employee work well-being (Zayid et al., 2024; Kinowska and Sienkiewicz, 2023) or explored its positive effect (Deng et al., 2025), with few studies simultaneously examining its positive and negative impacts. By explicitly considering job demands (time pressure and work overload) and job resources (feedback quality and role clarity) as distant mechanisms linking algorithmic control to well-being, our work explains why algorithmic control can be both positively and negatively linked to employee well-being. By doing so, it provides a comprehensive understanding of the influences of algorithmic control that go beyond the traditional view that algorithmic control is always detrimental. Second, the present study is an early application of the JD-R theory to algorithmic empirical research, which not only validates the propositions of the JD-R theory, but also enriches the literature on the antecedents of job demands and resources. Third, testing algorithmic control, extraversion, and neuroticism simultaneously in our model enables us to distinguish the unique predictive validity of algorithmic control.

## 2 Theoretical and hypotheses

### 2.1 Algorithmic control and JD-R theory

Existing studies have examined the effects of algorithmic control across diverse samples and work contexts. For instance, within the platform economy, research indicates that algorithmic control significantly influences food delivery drivers’ proactive customer service performance and customer-oriented service behaviors (Zhou et al., 2025; Yang et al., 2025). In traditional workplaces implementing algorithmic management, studies have found that algorithmic control can simultaneously promote employees’ knowledge hiding and stimulate their innovative behaviors (Liu et al., 2025a,b).

The JD-R theory states that management practices can simultaneously increase employees’ job resources and demands (Bakker et al., 2023). Job resources refer to “the psychological, physical, and social aspects of the job that are conducive to achieving work goals” (Demerouti et al., 2001, p. 501), such as job variety, role clarity, and feedback quality. Job demands refer to the “psychological, physical, and social aspects of the job that require sustained physical or mental effort and are therefore associated with certain physiological and psychological costs” (Demerouti et al., 2001, p. 501), such as work–family conflict, time pressure, and work overload. Furthermore, JD-R theory posits that job resources can motivate employees in terms of work engagement and generate more positive outcomes, whereas job demands can induce employee burnout and generate more negative outcomes (Bakker et al., 2023). Based on JD-R theory, Kloutsiniotis and Mihail (2020) suggested that high-performance work systems are in-directly related to employee burnout through the influences of job variety and work–family conflict. Drawing on the central tenets of JD-R theory, we argue that algorithmic control can simultaneously enhance employees’ job resources and job demands, which in turn affects the well-being of food delivery drivers. Specifically, on the one hand, algorithmic control provides delivery drivers with continuous, real-time, and frequent performance feedback through tracking evaluation, and standardized guidance (Pei et al., 2021), thereby enhancing the perceived specificity and timeliness of feedback and improving feedback quality. In addition, algorithmic control clearly delineates task scope, job responsibilities, and performance standards (Pei et al., 2021; Wu et al., 2023), which reduces role ambiguity and enhances role clarity. On the other hand, algorithmic control can implicitly or explicitly accelerate delivery drivers’ work pace through behavioral constraints and real-time monitoring (Pei et al., 2021; Zheng et al., 2019), thereby increasing time pressure. Moreover, continuous task allocation that gives limited consideration to drivers’ capacity may further increase the order volume assigned to food delivery drivers (Parent-Rocheleau and Parker, 2022), potentially resulting in work overload. Accordingly, in this study, time pressure and work overload are conceptualized as job demands, whereas feedback quality and role clarity are conceptualized as job resources.

Feedback quality, which forms an important aspect of the feedback environment, refers to the value and usefulness of feedback information (Steelman et al., 2004). High-quality feedback can provide accurate and useful guidance and assistance for employees when completing work tasks (Steelman et al., 2004). Thus, the quality of feedback can be considered a job resource (Peng and Chiu, 2010). Previous empirical research has proven that leader motivating language, leader learning goal orientation, and powerful artificial intelligence data analytics are important antecedents of feedback quality (Guo and Ling, 2020; Tong et al., 2021). Accordingly, because algorithmic control replaces the role of managers in traditional work (Duggan et al., 2020), we anticipate that algorithmic control is positively related to feedback quality.

First, feedback quality is enhanced through instructions from immediate supervisors, such as telling employees how to complete their work (Guo and Ling, 2020). As the direct supervisor of food delivery drivers, algorithmic control can automatically dispatch orders based on the geographical location of consumers, restaurants, and drivers. Furthermore, it can also plan the driver’s delivery route in advance, enabling them to avoid potential risks such as traffic accidents and traffic congestion during the delivery process (Wu et al., 2023). In this manner, these clear and timely algorithmic instructions can enhance the quality of feedback provided. Second, by providing drivers with real-time information regarding orders, customer evaluations, and daily income, algorithmic control enables them to obtain timely, accurate, and visual-based performance feedback information (Parent-Rocheleau and Parker, 2022; Huang, 2023). Feedback information of this nature simultaneously puts forward job requirements and provides helpful suggestions for drivers regarding how they can improve their future work performance (Parent-Rocheleau and Parker, 2022; Huang, 2023). In this way, algorithmic control renders performance feedback information more timely, accurate, helpful, and easy to understand, with these items forming the core of quality feedback (Steelman et al., 2004). Thus, we propose:

*H1*: Algorithmic control is positively related to feedback quality.

Role clarity is a job resource that refers to employees clearly knowing the tasks, objectives, and expectations associated with their jobs (Demerouti et al., 2012). A significant number of studies have explored the origins of role clarity, including regarding individual trait factors (i.e., internal work locus of control and general self-efficacy) and management styles (i.e., outcome-based managerial control, ethical leadership, and prevention-focused leadership) (Demerouti et al., 2012; Kauppila, 2014; Li and Griffin, 2022; Vullinghs et al., 2020). In the platform economy, algorithmic control plays the role of a line leader and provides direct guidance to food delivery drivers (Huang, 2023). Based on this, we argue that algorithmic control can enhance role clarity.

First, setting clear goals and helping employees clarify their task scope and job responsibilities play a crucial role in enhancing role clarity (Vullinghs et al., 2020; Rizzo et al., 1970). As the direct supervisor of food delivery drivers, algorithmic control not only provides personalized task goals and clarifies task scope through standardized guidance, but also monitors drivers’ work progress and service attitudes and delivers real-time feedback (Pei et al., 2021; Huang, 2023; Lu et al., 2024), thereby enabling drivers to develop a clearer understanding of their job responsibilities. Accordingly, algorithmic control enhances delivery drivers’ role clarity. Second, algorithmic control clarifies the rewards and punishments associated with different performance levels through behavioral constraints, thereby reducing ambiguity between work inputs and anticipated rewards and strengthening job expectations (Pei et al., 2021; Lu et al., 2024). Job expectations constitute a core component of role clarity (Vullinghs et al., 2020; Rizzo et al., 1970). Third, existing research indicates that direct and high-level supervision and guidance of employees’ work-related activities enhance role clarity (Kauppila, 2014). Algorithmic control, as a management practice that delivers comprehensive supervision and guidance through algorithmic systems, therefore plays a critical role in enhancing food delivery drivers’ role clarity. Thus, we propose:

*H2*: Algorithmic control is positively related to role clarity.

Time pressure is a job demand that refers to individuals not having enough time to complete their work, or facing the need to complete their work faster than usual (Ohly and Fritz, 2010). Employees facing time pressure clearly know that they need to put more effort into completing their work in a timely manner (Kern and Zapf, 2021). Drawing on JD-R theory, Yunus et al. (2023) demonstrated that high-performance HR practices are positively associated with employee job demands in the form of time demands. Dietz and Scheel (2017) showed that supervisorial pressure can increase the amount of time pressure experienced by employees. Therefore, in this study, we anticipated that algorithmic control would increase the time pressure faced by drivers.

First, algorithmic control quantitatively manages delivery drivers through stringent time-based performance indicators, such as order completion deadlines, on-time delivery rates, and average delivery durations. Drivers are required to complete their tasks within extremely tight time frames, and any delay may negatively affect their ratings and incentives (Yang et al., 2025; Huang, 2023). This exposes them to a constant sense of “countdown” and intensifies their experience of time pressure. Second, beyond merely tracking delivery progress, algorithmic control reinforces the salience of time through mechanisms such as real-time evaluations and ranking systems. This persistent control and surveillance not only require delivery drivers to devote additional time to coping with algorithmic demands (Zhou et al., 2025), but also cultivate a perception among drivers that time is equivalent to performance (Nguyen-Phuoc et al., 2023; Huang, 2023), thereby intensifying drivers’ perceived time pressure. Third, indirect empirical evidence suggests that algorithmically generated route planning significantly influences the time pressure experienced by drivers (Zheng et al., 2019). We therefore hypothesize:

*H3*: Algorithmic control is positively related to time pressure.

Work overload refers to an individual’s perception that their assigned work tasks exceed their own ability level (Moore, 2000). Employees who experience a high degree of work overload require additional work time and must put in more effort to complete their assigned tasks (Moore, 2000; Shang et al., 2023). As a job demand, work overload is more easily stimulated and influenced by high-performance work systems, prevention-focused leadership, and enterprise social media use (Kloutsiniotis and Mihail, 2020; Li and Griffin, 2022; Shang et al., 2023). Thus, we argue that algorithmic control is positively related to drivers’ experience of work overload.

First, algorithmic control assigns orders to drivers based on factors such as historical performance and the proximity of merchants and drivers, with limited consideration of drivers’ individual needs (Pei et al., 2021; Yang et al., 2025). This practice can result in an excessive allocation of tasks during high-demand periods (Yang et al., 2025), compelling drivers to complete multiple high-intensity tasks simultaneously and thereby increasing work overload. Moreover, algorithmic control categorizes food delivery drivers’ performance into hierarchical levels and ranks them within the platform, with these levels and rankings directly tied to compensation and rewards (Yang et al., 2025). This competitive system motivates drivers to continuously accept additional tasks in order to maintain a high rank or secure rewards (Lu et al., 2024), thereby exacerbating their work overload. In addition, although direct empirical evidence on the relationship between algorithmic control and work overload remains limited, prior studies have theoretically suggested that food delivery drivers are more likely to perceive heightened work overload under algorithmic control (Lu et al., 2024; Wu et al., 2023). We therefore hypothesize:

*H4*: Algorithmic control is positively related to work overload.

### 2.2 The relationship between job demands and well-being

Employee well-being is crucial to the survival and development of organizations (Spreitzer and Porath, 2012). As a multidimensional variable, researchers have suggested that the positive and negative dimensions of well-being should be considered simultaneously (Kaluza et al., 2021). In this study, we considered job satisfaction (frequently deemed an indicator of positive well-being; Puranik et al., 2021) and emotional exhaustion (frequently deemed an indicator of negative well-being; Caesens et al., 2017) as indicators of positive and negative well-being, respectively. Job satisfaction reflects a pleasurable emotional state that arises from making an overall evaluation of one’s job (Siu, 2002), whereas emotional exhaustion is defined as a type of burnout caused by work stressors (Maslach et al., 2001).

The JD-R theory states that high job demands consume individuals’ mental and cognitive resources, leading to resource depletion and burnout (Bakker et al., 2023). Therefore, drawing on JD-R theory, we argue that job demands (work overload and time pressure) can stimulate drivers’ emotional exhaustion and curb their level of job satisfaction. First, when delivery drivers experience a high level of work overload, they must expend additional individual resources to manage it successfully (Demerouti et al., 2001). Over time, this consumption of individual resources increases employees’ level of emotional exhaustion (Maslach et al., 2001), damages their psychological and physical health (Dlouhy and Casper, 2021), and reduces their job satisfaction. Second, empirical researches have demonstrated that work overload increases employees’ emotional exhaustion and reduces their level of job satisfaction (Hong et al., 2021; Shantz et al., 2016).

The JD-R theory also states that job demands can consume individual resources and reduce individual well-being (Bakker et al., 2023). To meet the requirements of algorithmic control, high time pressure delivery drivers must invest greater resources (i.e., working at a faster speed and violating traffic rules) to complete their delivery task within the estimated time (Nguyen-Phuoc et al., 2023), which will consume their self-control resources (Prem et al., 2017) and lead to emotional exhaustion (Nguyen-Phuoc et al., 2023). Moreover, when faced with high long-term levels of time pressure, delivery drivers find themselves in a prolonged state of overtaxing (Demerouti et al., 2001), which can trigger job-related anxiety and depression (Yunus et al., 2023), as well as reduced job satisfaction. Additionally, empirical studies have also found that job demands, including time pressure, can promote emotional exhaustion and reduce job satisfaction (Li et al., 2023; Krick et al., 2022). Therefore, we propose:

*H5a*: Work overload has a positive relationship with emotional exhaustion and a negative relationship with job satisfaction.

*H5b*: Time pressure has a positive relationship with emotional exhaustion and a negative relationship with job satisfaction.

The JD-R theory suggests that management practices influence employee well-being by reshaping job demands (Bakker et al., 2023). Accordingly, we propose that the influence of algorithmic control on emotional exhaustion and job satisfaction is indirectly transmitted through job demands (work overload and time pressure). That is, algorithmic control increases the amount of work overload and time pressure experienced by drivers (H3 and H4). This is subsequently expected to increase drivers’ level of emotional exhaustion and reduce their level of job satisfaction (H5). Thus, we hypothesize:

*H6a*: Work overload mediates the relationship between algorithmic control and well-being such that algorithmic control has a positive relationship with work overload, which, in turn, has a negative relationship with job satisfaction, and a positive relationship with emotional exhaustion.

*H6b*: Time pressure mediates the relationship between algorithmic control and well-being such that algorithmic control has a positive relationship with time pressure, which, in turn, has a negative relationship with job satisfaction, and a positive relationship with emotional exhaustion.

### 2.3 The relationship between job resources and well-being

According to JD-R theory, the provision of adequate job resources can promote employee growth and development, thereby increasing employee work engagement and reducing employee turnover intention (Bakker et al., 2023; Demerouti et al., 2001). Therefore, drawing on JD-R theory, we argue that job resources (feedback quality and role clarity) can enhance job satisfaction and suppress emotional exhaustion. Employees with a level of high role clarity have a clear understanding of their roles, tasks, and responsibilities at work, as well as the expectations and requirements of their supervisors (Kalshoven et al., 2011; Rizzo et al., 1970). They are also more likely to be satisfied with their job and experience a greater sense of job control (Vullinghs et al., 2020), which is beneficial for suppressing emotional exhaustion (Karasek, 1998). In the present study, drivers who perceived themselves as having a high level of role clarity displayed a strong understanding of their roles, job responsibilities, and algorithmic requirements in the context of order delivery services. They were also more likely to perceive a higher level of job satisfaction and control, and to experience less emotional exhaustion. Previous empirical evidence has also shown that role clarity is beneficial for enhancing employee job satisfaction and reducing job burnout (Vullinghs et al., 2020; Ritter et al., 2016), and that emotional exhaustion is an important dimension of job burnout (Halbesleben and Bowler, 2007).

Because high-quality feedback provides recipients with useful information relating to the completion of specific work tasks and the achievement of optimal job performance, recipients are more likely to perceive a high level of satisfaction, regardless of whether the feedback provided is positive or negative (Steelman et al., 2004). Accordingly, high-quality algorithmic feedback provides delivery drivers with specific and useful feedback information, such as regarding delivery routes, delivery times, and service performance (Huang, 2023; Wu et al., 2023). This kind of detailed feedback can stimulate drivers’ work satisfaction and reduce their level of emotional exhaustion (Wang et al., 2015; Nguyen-Phuoc et al., 2023). Furthermore, indirect empirical researches have repeatedly found that the feedback environment, which also encompasses feedback quality, has a positive effect on improving job satisfaction and suppressing job burnout (Sparr and Sonnentag, 2008; Gong et al., 2017). Therefore, we propose:

*H7a*: Role clarity has a positive relationship with job satisfaction and a negative relationship with emotional exhaustion.

*H7b*: Feedback quality has a positive relationship with job satisfaction and a negative relationship with emotional exhaustion.

The JD-R theory suggests that management practices influence employee well-being by reshaping job resources (Bakker et al., 2023). Accordingly, we propose that the influence of algorithmic control on emotional exhaustion and job satisfaction is indirectly transmitted through job resources (role clarity and feedback quality). That is, algorithmic control increases the amount of role clarity and feedback quality experienced by drivers (H1 and H2). This is subsequently expected to increase drivers’ level of job satisfaction and reduce their level of emotional exhaustion (H7). Thus, we hypothesize:

*H8a*: Feedback quality mediates the relationship between algorithmic control and well-being such that algorithmic control has a positive relationship with feedback quality, which, in turn, has a positive relationship with job satisfaction, and a negative relationship with emotional exhaustion.

*H8b*: Role clarity mediates the relationship between algorithmic control and well-being such that algorithmic control has a positive relationship with role clarity, which, in turn, has a positive relationship with job satisfaction, and a negative relationship with emotional exhaustion.

**Figure 1:**
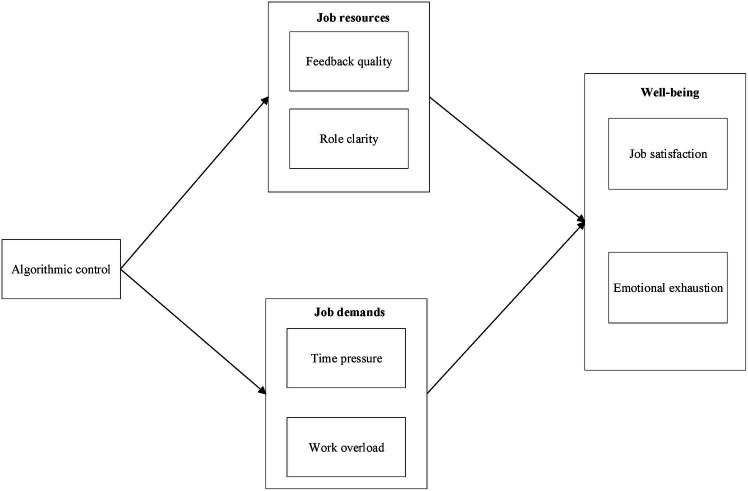
Conceptual model.

In summary, the conceptual model proposed in this study is illustrated in Figure 1.

## 3 Method

### 3.1 Sample

The sample for this study comprised delivery drivers affiliated with Meituan and Ele.me platforms in Chongqing, China. Meituan and Ele.me are the two largest food delivery platforms in China, with 7.45 million and 4 million delivery drivers, respectively. With the assistance of station managers from both firms, we introduced the purpose of this study and the corresponding data collection procedures to 702 delivery drivers, of whom 687 were willing to participate. A three-stage questionnaire collection method was adopted to reduce the impact of common method variances (CMV). Specifically, at Time 1, 592 (86.17% response rate) delivery drivers completed questionnaires that addressed the topics of algorithmic control, extraversion, neuroticism, and demographic variables. At Time 2 (1 month after Time 1), 527 (76.71% response rate) delivery drivers responded to questionnaires designed to measure the extent of their job demands (time pressure and work overload) and job resources (feedback quality and role clarity). At Time 3(1 month after Time 2), 446 delivery drivers provided information relating to their own well-being (job satisfaction and emotional exhaustion).

### 3.2 Measures

Variables in the delivery driver surveys were asked to indicate the extent to which they agreed with the statements on a 5-point Likert scale ranging from 1 (strongly disagree) to 5 (strongly agree).

Algorithmic control. Due to the focus of this study on food delivery drivers, we used Pei et al.’s (2021) eleven items to measure algorithmic control. A sample item is “Algorithm intelligently assigns my work tasks” (*α* = 0.95).

Feedback quality. We used Steelman et al.’s (2004) five items to measure feedback quality. A sample item is “Algorithms give me useful feedback on my job performance” (α = 0.91).

Role clarity. We used Rizzo et al.’s (1970) five items to measure role clarity. A sample item is “I have clear and planned objectives for my work” (α = 0.85).

Time pressure. We used Durham et al.’s (2000) four items to measure time pressure. A sample item is “Task durations are often short” (α = 0.84).

Work overload. We used Moore’s (2000) four items to measure work overload. A sample item is “I feel that I have handled more requests, questions or complaints than expected” (α = 0.84).

Job satisfaction. We used Warr et al.’s (1979) three items to measure job satisfaction. A sample item is “Overall, I’m satisfied with my current job” (α = 0.92).

Emotional exhaustion. We used Schaufeli et al.’s (1996) five items to measure emotional exhaustion. A sample item is “I feel exhausted at the end of the working day” (α = 0.87).

Control variables. First, marriage (0 = unmarried, 1 = married) was controlled because it can provide emotional support to individuals (Zhang et al., 2022). Second, previous research suggests that education is beneficial in helping employees cope with changes in the workplace (Zhang et al., 2022). Thus, we controlled for education (1 = employees held high school diploma or below, 2 = employees held junior college degree, 3 = employees held bachelor degree, 4 = employees held postgraduate degree or above). Third, gender (1 = female, 2 = male) was included since there are differences in well beings among employees of different genders (Kaluza et al., 2021). Fourth, employees across different age groups(1 = under 25 years old, 2 = 25–35 years old (excluding 35 years old), 3 = 35–45 years old (excluding 45 years old), 4 = 45 years old and above) and position tenure(1 = less than 1 year, 2 = 1–5 years (excluding 5 years), 3 = 5–10 years (excluding 10 years), 4 = 10 years and above) may show different levels of well-being due to factors such as vigor and health (Zhang et al., 2022). Thus, we controlled for drivers’ age and position tenure. In addition, previous researches have found that extraversion has a positive effect on employee well-being, while neuroticism has a negative impact on employee well-being (Anglim and Horwood, 2021). Therefore, we controlled for extra-version and neuroticism. We used Caci et al.’s (2014) four items to measure extraversion, a sample item is “I like to have a lot of people around me” (α = 0.88). Neuroticism is also measured by four items as developed by Caci et al. (2014), a sample item is “I feel unable to manage different situations” (α = 0.88).

## 4 Results

### 4.1 Confirmatory factor analysis (CFA)

Because the data on algorithmic control, feedback quality, role clarity, time pressure, work overload, job satisfaction, emotional exhaustion, neuroticism and extraversion were collected from delivery drivers, we use AMOS 26.0 maximum likelihood estimation to conduct the CFA. The results (Table 1) show that the proposed nine-factor model (χ^2^ = 1798.33, *df* = 909, *p* < 0.001; RMSEA = 0.05, SRMR = 0.05, AIC = 2050.33, CFI = 0.93, IFI = 0.93) was a significantly better fit than an eight-factor model (i.e., combing neuroticism and extraversion into one factor, χ^2^ = 2727.36, *p* < 0.001; RMSEA = 0.07, SRMR = 0.08 AIC = 2963.36, CFI = 0.85, IFI = 0.85), a seven-factor model (i.e., combing neuroticism, emotional exhaustion and extraversion into one factor, χ^2^ = 3653.63, *p* < 0.001; RMSEA = 0.08, SRMR = 0.09, AIC = 3875.63, CFI = 0.77, IFI = 0.77), a six-factor model (i.e., combing neuroticism, job satisfaction, emotional exhaustion and extraversion into one factor, χ^2^ = 4574.32, *p* < 0.001; RMSEA = 0.10, SRMR = 0.10, AIC = 4784.32, CFI = 0.70, IFI = 0.70), a five-factor model (i.e., combing neuroticism, work overload, job satisfaction, emotional exhaustion and extraversion into one factor, χ^2^ = 5195.09, *p* < 0.001; RMSEA = 0.10, SRMR = 0.11, AIC = 5385.09, CFI = 0.64, IFI = 0.62), a four-factor model (i.e., combing neuroticism, time pressure, work overload, job satisfaction, emotional exhaustion and extraversion into one factor, χ^2^ = 5837.99, *p* < 0.001; RMSEA = 0.11, SRMR = 0.12, AIC = 6029.99, CFI = 0.59, IFI = 0.52), a three-factor model (i.e., combing neuroticism, role clarity, time pressure, work overload, job satisfaction, emotional exhaustion and extraversion into one factor, χ^2^ = 6555.19, *p* < 0.001; RMSEA = 0.12, SRMR = 0.13, AIC = 6741.19, CFI = 0.53, IFI = 0.53), a two-factor model (i.e., combing neuroticism, role clarity, time pressure, work overload, job satisfaction, feedback quality, emotional exhaustion and extraversion into one factor, χ^2^ = 7173.82, *p* < 0.001; RMSEA = 0.12, SRMR = 0.13, AIC = 7355.82, CFI = 0.48, IFI = 0.48) and a one-factor model (χ^2^ = 9287.54, *p* < 0.001; RMSEA = 0.14, SRMR = 0.18, AIC = 9467.54, CFI = 0.30, IFI = 0.31), confirming discriminant validity. Furthermore, Table 2 shows that the composite reliability (CR) and average variance extracted (AVE) for all variables were greater than 0.8 and 0.5, respectively, supporting convergent validity.

**Table 1:** Confirmatory factor analyses results.

Model	χ^2^	*df*	Δχ^2^	Δ*df*	RMSEA	SRMR	AIC	CFI	IFI
Nine-factor model	1798.33	909	–	–	0.05	0.05	2050.33	0.93	0.93
Eight-factor model	2727.36	917	929.03^***^	8	0.07	0.08	2963.36	0.85	0.85
Seven-factor model	3653.63	924	1855.30^***^	15	0.08	0.09	3875.63	0.77	0.77
Six-factor model	4574.32	930	2775.99^***^	21	0.10	0.10	4784.32	0.70	0.70
Five-factor model	5195.09	935	3396.76^***^	26	0.10	0.11	5385.09	0.64	0.62
Four-factor model	5837.99	939	4039.66^***^	30	0.11	0.12	6029.99	0.59	0.52
Three-factor model	6555.19	942	4756.86^***^	33	0.12	0.13	6741.19	0.53	0.53
Two-factor model	7173.82	944	5375.49^***^	35	0.12	0.13	7355.82	0.48	0.48
One-factor model	9287.54	945	7489.21^***^	36	0.14	0.18	9467.54	0.30	0.31

“+” represents the two factors were combined into one factor. *** denotes *p* < 0.001.

AC, Algorithmic control; FQ, Feedback quality; RC, Role clarity; TP, Time pressure; WO, Work overload; JS, Job satisfaction; EE, Emotional exhaustion; NE, Neuroticism; EX, Extraversion.

Nine-factor model = AC, FQ, RC, TP, WO, JS, EE, NE, EX; Eight-factor model = NE+EX, AC, FQ, RC, TP, WO, JS, EE; Seven-factor model = NE+EX+EE, AC, FQ, RC, TP, WO, JS; Six-factor model = NE+EX+EE+JS, AC, FQ, RC, TP, WO; Five-factor model = NE+EX+EE+JS +WO, AC, FQ, RC, TP; Four-factor model = NE+EX+EE+JS +WO+TP, AC, FQ, RC; Three-factor model = NE+EX+EE+JS +WO+TP +RC, AC, FQ; Two-factor model = E + EX+EE+JS +WO+TP +RC+FQ, AC; One-factor model = All items loaded on one factor.

**Table 2:** Means, standard deviations, and correlations.

Variable	Mean	SD	1	2	3	4	5	6	7	8	9	10	11	12	13	14
1. Genders	1.73	0.44	1													
2. Age	2.58	0.81	−0.10	1												
3. Marriages	0.63	0.49	−0.10^*^	0.18^**^	1											
4. Education attainment	1.81	0.82	0.04	0.07	0.02	1										
5. Position tenure	2.94	0.84	0.07	0.02	−0.29^**^	0.05	1									
6. Algorithmic control	3.40	0.65	0.08	0.09^*^	−0.06	0.04	0.04	1								
7. Role clarity	3.29	0.68	−0.01	0.16^**^	−0.06	0.04	0.07	0.21^**^	1							
8. Neuroticism	3.08	0.78	−0.01	0.05	−0.01	0.12^*^	0.06	0.07	−0.05	1						
9. Work overload	3.14	0.73	0.05	−0.01	−0.06	−0.09^*^	0.03	0.19^**^	−0.09	0.10^*^	1					
10. Time pressure	3.42	0.70	0.01	0.05	−0.08	−0.07	0.09	0.23^**^	−0.12^**^	0.07	0.14^**^	1				
11. Job satisfaction	3.14	0.88	0.04	0.002	−0.06	0.06	−0.04	0.22^**^	0.29^**^	−0.14^**^	−0.23^**^	−0.21^**^	1			
12. Feedback quality	3.25	0.77	0.07	0.004	−0.10^*^	0.02	0.04	0.18^**^	0.23^**^	−0.09	−0.11^*^	−0.12^*^	0.50^**^	1		
13. Extroverted	3.21	0.74	0.002	−0.03	0.06	−0.02	−0.03	0.13^**^	0.17^**^	−0.04	−0.12^*^	−0.05	0.26^**^	0.40^**^	1	
14. Emotional exhaustion	3.09	0.71	0.06	0.04	0.12*	0.02	0.04	0.04	−0.21^**^	0.26^**^	0.24^**^	0.24^**^	−0.18^**^	−0.30^**^	−0.22^**^	1
AVE	–	–	–	–	–	–	–	0.61	0.54	0.65	0.57	0.58	0.80	0.66	0.65	0.57
CR	–	–	–	–	–	–	–	0.95	0.85	0.88	0.84	0.84	0.92	0.91	0.88	0.87

Gender: 1 = female, 2 = male; Age: 1 = under 25 years old, 2 = 25–35 years old (excluding 35 years old), 3 = 35–45 years old (excluding 45 years old), 4 = 45 years old and above. Marriages: 0 = unmarried, 1 = married. Education: 1 = employees held high school diploma or below, 2 = employees held junior college degree, 3 = employees held bachelor degree, 4 = employees held postgraduate degree or above. Tenure: 1 = less than 1 year, 2 = 1–5 years (excluding 5 years), 3 = 5–10 years (excluding 10 years), 4 = 10 years and above. AVE = average variance extracted, CR = composite reliability. * denotes *p* < 0.05, ** denotes *p* < 0.01.

### 4.2 Common method variance (CMV)

First, the Harman’s single-factor analysis results indicate that the first factor explained 19.16% of the total variance. Second, we test a ten-factor model that included the nine-factor model (algorithmic control, feedback quality, role clarity, time pressure, work overload, job satisfaction, emotional exhaustion, neuroticism, and extraversion) and a CMV factor. The results showed that compared to the nine-factor model, the ten-factor model resulted in a significant improvement in model fit (Δχ^2^ [45] = 281.082, *p* < 0.001). However, the variance extracted by the CMV factor was only 2.38%, less than the suggested floor of 25% (Hoobler and Hu, 2013). Third, Table 2 shows that the correlation coefficients of all variables are less than or equal to 0.50. Therefore, the CMV in the present study is not a serious concern.

### 4.3 Descriptive statistics and correlations

Table 2 reveals that algorithmic control was positively correlated with feedback quality (*r* = 0.18, *p* < 0.01), role clarity (*r* = 0.21, *p* < 0.01), time pressure (*r* = 0.23, *p* < 0.01), and work overload (*r* = 0.19, *p* < 0.01). Furthermore, feedback quality was negatively correlated with emotional exhaustion (*r* = −0.30, *p* < 0.01) and positively correlated with job satisfaction (*r* = 0.50, *p* < 0.01); role clarity was negatively correlated with emotional exhaustion (*r* = −0.21, *p* < 0.01) and positively correlated with job satisfaction (*r* = 0.29, *p* < 0.01); time pressure was positively correlated with emotional exhaustion (*r* = 0.24, *p* < 0.01) and negatively correlated with job satisfaction (*r* = −0.21, *p* < 0.01); and work overload was positively correlated with emotional exhaustion (*r* = 0.24, *p* < 0.01) and negatively correlated with job satisfaction (*r* = −0.23, *p* < 0.01).

### 4.4 Hypotheses testing

**Figure 2:**
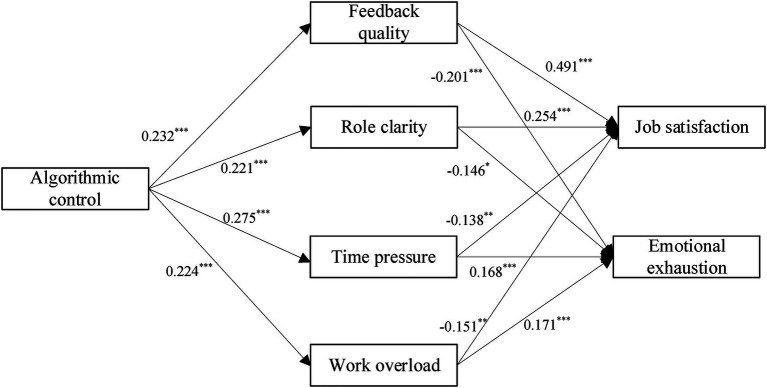
Unstandardized path coefficients for the proposed model.

Our research deploys structural equation modeling with AMOS 26.0 maximum likelihood estimation to examine our hypotheses. The structural model showed an acceptable fit (χ^2^ = 2225.46, *df* = 1,125, *p* < 0.001; RMSEA = 0.047, CFI = 0.91, SRMR = 0.08) (Kline, 2022). As seen in Figure 2, the path coefficient between algorithmic control and feedback quality (*b* = 0.232, *p* < 0.001) was positive and significant, supporting Hypotheses 1. Further, the path coefficient between algorithmic control and role clarity (*b* = 0.221, *p* < 0.001) was positive and significant, supporting Hypotheses 2. Additionally, algorithmic control was positively related to time pressure (*b* = 0.275, *p* < 0.001) and work overload (*b* = 0.224, *p* < 0.001), supporting Hypotheses 3 and Hypotheses 4.

In support of Hypotheses 5a, Figure 2 showed that work overload had a positive relationship with emotional exhaustion (*b* = 0.171, *p* < 0.001), but had a negative relationship with job satisfaction (*b* = −0.151, *p* < 0.01). In support of Hypotheses 5b, Figure 2 showed that time pressure had a positive relationship with emotional exhaustion (*b* = 0.168, *p* < 0.001), but had a negative relationship with job satisfaction (*b* = −0.138, *p* < 0.01). In support of Hypotheses 7a, Figure 2 showed that role clarity had a negative relationship with emotional exhaustion (*b* = −0.146, *p* < 0.05), but had a positive relation-ship with job satisfaction (*b* = 0.254, *p* < 0.001). In support of Hypotheses 7b, Figure 2 revealed that feedback quality had a negative relationship with emotional exhaustion (*b* = −0.201, *p* < 0.001), but had a positive relationship with job satisfaction (*b* = 0.491, *p* < 0.001).

Regarding the mediation hypotheses, we followed the recommendation outlined by Preacher and Hayes (2008) by using AMOS 26.0. Specifically, we estimated them by using a user-defined estimated called MyIndirectEffects for AMOS 26.0 (Lee et al., 2024) with 95% confidence intervals and 5,000 bootstrap samples. The mediation effect is considered significant when the 95 percent confidence intervals fail to cover zero. The results showed that the effect of algorithmic control on job satisfaction (*b* = −0.034, CI = −0.083 to −0.008) and emotional exhaustion (*b* = 0.038, CI = 0.007 to 0.095) was mediated by work overload, supporting Hypothesis 6a. In support of Hypothesis 6b, the effect of algorithmic control on job satisfaction (*b* = −0.038, CI = −0.09 to −0.008) and emotional exhaustion (*b* = 0.046, CI = 0.013 to 0.103) was mediated by time pressure. In addition, the effect of algorithmic control on job satisfaction (*b* = 0.114, CI = 0.035 to 0.220) and emotional exhaustion (*b* = −0.047, CI = −0.115 to −0.008) was mediated by feedback quality, supporting Hypothesis 8a. In support of Hypothesis 8b, the effect of algorithmic control on job satisfaction (*b* = 0.056, CI = 0.016 to 0.127) and emotional exhaustion (*b* = −0.032, CI = −0.081 to −0.006) was mediated by role clarity.

## 5 Discussion

### 5.1 Theoretical implications

First, drawing on JD-R theory, this study enriches the existing research in this area by establishing a dual-pathway model of the relationship between algorithmic control and well-being. Most of the existing researches have been based on self-determination theory and conservation of resources theory, which suggest that algorithmic control suppresses employee work well-being by reducing job autonomy and enhancing job burnout (Zayid et al., 2024; Kinowska and Sienkiewicz, 2023), they have overlooked the double-edged sword effect that algorithmic control may have on employees’ well-being. By simultaneously investigating both the salutary and detrimental effects of algorithmic control, our research findings have demonstrated that algorithmic control may enhance drivers’ emotional exhaustion and suppress their job satisfaction through its association with high job demands (work overload and time pressure). More importantly, this study also supports the positive effects of algorithmic control. Our results demonstrate the validity of the intriguing prediction that algorithmic control curbs emotional exhaustion and stimulates job satisfaction by increasing job resources (role clarity and feedback quality). This not only validates the theoretical viewpoint proposed by researchers that algorithmic control has a double-edged sword effect on employee outcomes (Parent-Rocheleau and Parker, 2022; Wu et al., 2023), but also provides a new theoretical perspective for exploring the double-edged sword effect of algorithmic control in the future.

Second, this study contributes to the JD-R theory. The JD-R theory has been applied in many domains (i.e., ethnicity, downsizing, and age discrimination) (Yeung et al., 2021; Dlouhy and Casper, 2021; Adamovic et al., 2022). Nevertheless, to the best of our knowledge, the present study is an early application of the JD-R theory to the algorithmic context, mapping the opposing mechanisms between algorithmic control and well-being. Although Huo et al. (2025) explored the direct inverted U-shaped effect of algorithmic control on employee work well-being based on the JD-R theory, they did not examine the potential mediators between algorithmic control and employee well-being. According to the perspective of the JD-R theory that management practices can simultaneously stimulate both job demands and resources, this research further demonstrates that the JD-R theory can be used in the algorithmic context. Moreover, while previous researches have mainly focused on situation factors (i.e., downsizing, age discrimination, and paternalistic leadership) that affect job demands and resources (Yeung et al., 2021; Dlouhy and Casper, 2021; Adamovic et al., 2022; Lee et al., 2024), with few studies analyzing the antecedents of job demands and resources from the perspective of algorithmic control. Our research shows that algorithmic control can simultaneously induce drivers’ job demands (work overload and time pressure) and job resources (role clarity and feedback quality), expanding the research on the antecedents of the job demands and resources.

Third, this study provides empirical evidence supporting the unique power that algorithmic control has on drivers’ well-being beyond other personality traits. Previously established independent variables, including extraversion and neuroticism (Garcia and Erlandsson, 2011; Anglim and Horwood, 2021), are useful for understanding the antecedents of employee well-being. By controlling for the effects of extraversion and neuroticism, this study was able to empirically test the unique predictive power of algorithmic control. In this way, the results obtained not only provide support for the predictive validity of algorithmic control for predicting drivers’ well-being in the form of emotional exhaustion and job satisfaction in the presence of extraversion and neuroticism, but also identify the opposing transmission mechanisms, as noted above.

### 5.2 Practical implications

Our findings indicate that algorithmic control may reduce delivery drivers’ well-being by increasing the job demands they experience (i.e., time pressure and work overload). Thus, it is necessary to remind platform firms and managers regarding the pitfalls of algorithmic control and to encourage them to take measures to reduce the impact of these negative effects. For example, platform firms can optimize algorithm rules by extending delivery times and improving evaluation systems, thereby reducing the job demands of delivery drivers. Another important point to note is that delivery firms have set up fixed stations and station managers in each city. The job responsibilities of these station managers include providing training and safety guidance for delivery drivers, sharing experiences of how to avoid negative customer reviews, and handling appeal matters for delivery drivers, with these measures helping to make up for the shortcomings of the algorithmic control process (Wu et al., 2023). In this way, station managers can provide job resources for delivery drivers by demonstrating benevolent and service-oriented leadership behaviors, thereby compensating for the high-level job demands induced by algorithmic control. The results of our study also indicate that algorithmic control may enhance delivery drivers’ well-being by increasing their level of job resources (feedback quality and role clarity). Thus, platform firms can not only enrich the feedback information provided and optimize information quality based on the needs of food delivery drivers but also enhance their trust in algorithmic control by explaining the rules of algorithmic design. Ultimately, this can maximize the positive role that algorithmic control plays in enhancing the job resources of food de-livery drivers.

### 5.3 Limitations and future directions

First, the use of a sample of delivery drivers limits the generalizability of these research findings with regard to other types of gig workers. Future research could use Uber and DiDi drivers for their samples to further test the validity of the theoretical model constructed in this study. Second, while this study conceptualizes algorithmic control as a holistic construct and demonstrates its dual effects on employee well-being, algorithmic control comprises three distinct dimensions, namely standardized guidance, tracking evaluation, and behavioral constraint, each of which may exert differential effects on employees (Zhou et al., 2025). Accordingly, future research could simultaneously examine the specific mechanisms through which each dimension of algorithmic control influences employee well-being, while identifying the key job demands and job resources that mediate these effects. Third, although the independent variable (algorithmic control), mediating variables (time pressure, work overload, feedback quality, and role clarity), and dependent variables (job satisfaction and emotional exhaustion) were collected from delivery drivers at different time points, causal inferences cannot be drawn from the cross-sectional nature of the data. Future research could build on prior studies such as Ozcan et al. (2023) and Shao et al. (2025) and employ experimental designs or experience sampling methods to more rigorously test the causal relationships. Fourth, this study incorporated eight mechanisms and did not consider the potential moderating effect of algorithmic control on job demands/resources. Future research can consider station managers’ behavior and drivers’ resources as moderators. Fifth, this study analyzes data from food delivery drivers working for two Chinese food delivery platforms embedded in a high power distance cultural context. In such contexts, delivery drivers are more likely to perceive algorithmic control as a legitimate and taken-for-granted form of managerial authority (Liu et al., 2025b; Şahin et al., 2021), which fosters acceptance of algorithmic control and, in turn, attenuates its negative effects. By contrast, in low power distance cultural contexts such as the United States, delivery drivers are more likely to perceive algorithmic control as an externally imposed and coercive form of control, which may elicit resistance and thereby amplify its negative effects. Accordingly, future research could re-examine the proposed theoretical model in low power distance cultural contexts to assess the generalizability of the present findings.

## 6 Conclusion

Drawing on JD-R theory, this research enriched our understanding of the relationship between algorithmic control and delivery drivers’ well-being by clarifying the underlying mediating mechanism of job resources (feedback quality and role clarity) and job demands (time pressure and work overload). Hopefully, our research findings may use as an impetus for future research into the double-edged sword effect of algorithmic control.

## Data Availability

The raw data supporting the conclusions of this article will be made available by the authors, without undue reservation.
